# Meta-Analysis and Systematic Review of HLA DQ2/DQ8 in Adults with Celiac Disease

**DOI:** 10.3390/ijms24021188

**Published:** 2023-01-07

**Authors:** Sara Aboulaghras, Daniela Piancatelli, Khalid Taghzouti, Abdelaali Balahbib, Mohammed Merae Alshahrani, Ahmed Abdullah Al Awadh, Khang Wen Goh, Long Chiau Ming, Abdelhakim Bouyahya, Khadija Oumhani

**Affiliations:** 1 Physiology and Physiopathology Team, Faculty of Sciences, Genomic of Human Pathologies Research, Mohammed V University, Rabat 10100, Morocco; 2 Laboratoire Immunologie, Institut National Hygiene, Rabat 10000, Morocco; 3 National Research Council (CNR)-Institute of Translational Pharmacology (IFT), 67100 L’Aquila, Italy; 4 Laboratory of Zoology and General Biology, Faculty of Sciences, Mohammed V University in Rabat, Rabat 10100, Morocco; 5 Department of Clinical Laboratory Sciences, Faculty of Applied Medical Sciences, Najran University, Najran 61441, Saudi Arabia; 6 Faculty of Data Science and Information Technology, INTI International University, Nilai 71800, Malaysia; 7 School of Medical and Life Sciences, Sunway University, Sunway City 47500, Malaysia; 8 Laboratory of Human Pathologies Biology, Department of Biology, Faculty of Sciences, Mohammed V University, Rabat 10100, Morocco

**Keywords:** celiac disease, HLA DQ2, HLA DQ8, adult, systematic review

## Abstract

Although people with human leukocyte antigens (HLA) DQ2 and/or DQ8 are more likely to develop celiac disease (CD), the condition cannot be fully explained by this genetic predisposition alone. Multiple, as yet unidentified, factors contribute to the genesis of CD, including genetics, the environment, and the immune system. In order to provide insight into a prospective possibility and an expanded screening technique, we aim to undertake a comprehensive and meta-analytical study of the assessment and distribution of HLA class II (HLA-DQ2/DQ8) in adult CD patients. A systematic review was conducted using an electronic search of databases (PubMed, Google Scholar, Embase, and Direct Science) from January 2004 to February 2022. DQ2/DQ2 homozygotes have the highest risk of developing CD. DQ2/DQ8 typing is an effective test to exclude CD from the differential diagnosis of a patient with CD symptoms. Although other non-HLA genes have been associated with CD, they are rarely considered at diagnosis because they account for only a small proportion of the heritability of CD. This finding, together with the information gathered previously, may be useful in considering widely available and economically feasible screening options for celiac disease in young people.

## 1. Introduction

Celiac disease is an autoimmune disorder triggered by eating gluten, a protein essential to wheat, barley, and rye, in those who are susceptible to it because of their genes [1,2]. CD has a high heritability of 75%. However, the non-HLA heritability is just 68%. The remaining CD variability is attributed to both shared (17%) and non-shared (15%) environmental variables [3]. The leukocyte histocompatibility antigen genes HLA-DQ2 encoded by (HLA-DQA1*05-DQB1*02) and HLA DQ8 encoded by (DQA1*03-DQB1*0302) located on chromosome 6p21 are the most important genes for the predisposition to this disorder [4]. Most celiac disease patients express the HLA-DQ2 haplotype (90%), around 5% of CD patients express the HLA-DQ8 haplotype, and almost 5% of the remaining individuals carry at least one of the two genes [5]. CD’s prevalence varies by region, but estimates place it between 0.5 and 1% worldwide [6,7]. CD seems to be more prevalent in patients suffering from autoimmune diseases and genetic diseases than it is among healthy persons.

In the United States, the rate of CD prevalence is 0.71% (1 in 141 people), which is on par with the rates seen in some European nations. CD is more common in Finland and Sweden, where the rate is 2–10%, than in Germany, where it is just 0.20% [8]. Most instances, however, go undetected. This frequency varies with age, geographical area, and gender. Serological testing found a prevalence of 1.4% for celiac disease, whereas biopsy testing found a frequency of 0.7% [9,10]. Analysis of CD prevalence in four regions (Oceania, the Middle East, East Asia, and South Asia) revealed that South Asia had the greatest incidence of CD (0.8%) and (7.1%) among low- and high-risk populations, respectively. The highest seroprevalence of CD (1.4%), however, is seen in Middle Eastern nations [11].

It is believed that the prevalence rate in Africa is around 1.1%. In point of fact, the frequency of the HLA-DQ2 haplotype in the population, as well as the consumption of wheat, is greater in North Africa than it is in sub-Saharan Africa [12]. Patients who suffer from celiac disease almost always have abnormal intestinal permeability [13]. Increased intestinal permeability was more common among celiac disease patients with active illness compared to those in remission [14]. Despite the growing non-classical appearance, micronutrient deficiencies are nevertheless frequent in individuals with CD [15]. However, this multi-systemic disorder is also present in the asymptomatic form that has been shown in particular in adults who tend to remain asymptomatic or oligosymptomatic [16]. Over the last two decades, however, the presentation of celiac disease in adults has shifted, and now, in 60% of instances, the illness manifests in extra-digestive forms [17].

Our goal is to perform a meta-analysis of the study of HLA class II human leukocyte antigens (HLA-DQ2/DQ8) in adult patients with CD to provide light on a possible viewpoint and method for broader screening. In contrast to several other reviews, we are only interested in material pertaining to CD and not gluten intolerance or sensitivity [18].

## 2. Research Methodology

### 2.1. Methods

We did an extensive search on the Google Scholar, Science Direct, Pub Med, Embase, and Medline databases using the following keywords (HLA OR HLADQ antigens OR HLA OR HLADQ antigens OR human leukocyte antigen (leukocyte OR leukocytes) AND (antigen OR antigens)) AND (HLADQ2 OR HLADQ8 OR HLADQ2 and HLADQ8) OR ((celiac OR celiac disease OR CD) AND (HLA OR HLADQ antigens OR adult OR adults)). Our search was restricted to the English language, and one of the topic phrases we used was “celiac illness.” The topic terms of the technique for finding the frequency of predisposing genes were “HLA antigens,” “class II histocompatibility antigens,” or “major histocompatibility complex antigens,” and the text words were “DQ2” and “DQ8.” A manual search of the references of the studies whose complete texts were examined was another method that was used to locate the papers. When searching for publication, time, status, or the language of publishing, no constraints were used. Abstracts that were not also made available in full-text format were not considered for inclusion in this research.

We conducted a meta-analysis of all available literature on the topic of HLA DQ and Celiac disease in adults.

A.S. and K.O. both did the literature evaluation, read all of the entire texts, and independently selected which studies to include and which to exclude based on the inclusion and exclusion criteria. A discussion was used to iron out any differences of opinion between the 2 writers. In the event of a prolonged dispute, the senior member of the team (K.T) would go through the data and make a decision.

#### 2.1.1. The Selection Criteria

Prior to beginning the literature search, we established what would be considered included and what would be disqualified. Studies that matched the following criteria and were published as full articles or abstracts were included in this meta-analysis:

1. The study must be designed for adults CD patients;
2. Studies involving patients with CD and not gluten sensitivity;
3. The study sample must be representative—the number of participants must be greater than 30;
4. The test subjects may be related or unrelated and include both men and women;
5. The data must include the haplotype distributions in terms of their frequency HLA DQ2 HLA DQ8.

#### 2.1.2. The Exclusion Criteria

1. Studies Treating Celiac Disease in Association with Another Disease;
2. Studies that focus only on children;
3. This includes many versions of the same article, letter, editorial, case series, narrative review, systematic review, or meta-analysis. Following the title and/or reading an abstract, we found that there were many articles that did not pertain to our topic;
4. We also don’t include studies that don’t help us answer our research question.

#### 2.1.3. Quality of Studies

The quality of the included studies was independently assessed by 2 reviewers (S.A, K.O) using a checklist designed by Penny Whiting (Table 1) using the QUADAS (Diagnostic Accuracy Studies) tool for diagnostic accuracy studies [19]. This tool allows the assessment of the quality of observational studies. Due to the complexity of interpreting the results and the loss of information that would result from aggregating the scores from the 14 questions (to which respondents might respond “yes,” “no,” or “unclear”), we are unable to provide a summary score for this assessment. The point system attempts to show whether or not the patients in the study are representative of those receiving the test in practice; the accuracy and consistency of the reference standard; the appropriateness of the time between the standard and the test being evaluated; the interpretation of results; the explanation; and the synthesis and analysis of data. In cases where disagreements developed, the 2 reviewers ultimately agreed on this evaluation.

#### 2.1.4. Data Extraction

Both researchers (A.S. and K.O.) critically reviewed the included studies to extract the pertinent data separately, and any disagreements were settled by consensus. Author, year published, the year studied, country studied, population characteristics, study type, population characteristics (number of participants, percentage of males, percentage of women, and age of participants), and diagnosis were all retrieved.

## 3. Statistical Analysis

For the meta-analysis, we performed the statistical analysis using the R studio software (Version 4.1.3, University of Auckland, Auckland, New Zealand). The random effect model was used for the analysis. We calculated the odds ratios (OR) and 95% confidence intervals (CI).

Heterogeneity was tested with I^2^- and chi^2^-heterogeneity. An I^2^ of 0–40%, 30–60%, 50–90%, and 75–100% represents heterogeneity between studies that are not significant, moderate, substantial, and considerable, with *p* < 0.10 indicating statistical significance.

The data were analyzed using Statistical Package for Social Science (SPSS) version 21. The frequencies of the clinical and histological characteristics were calculated as part of a descriptive analysis.

## 4. Results

### Procedures for Identifying and Selecting Studies

**Figure 1:**
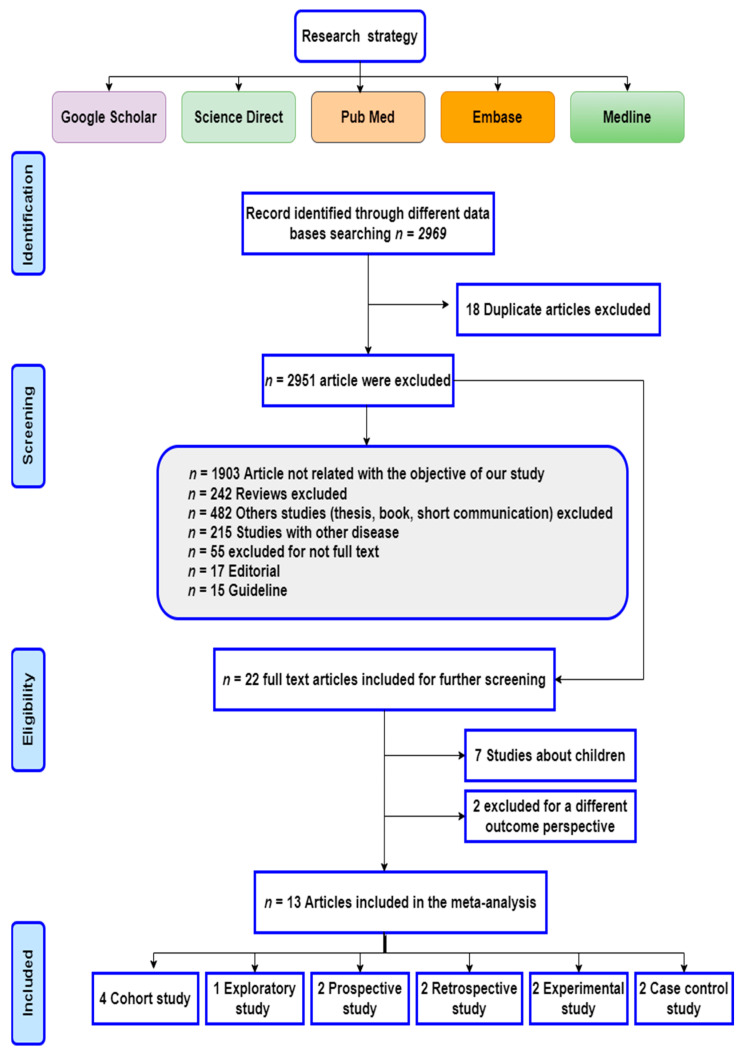
Flow diagram of the study following the PRISMA statement.

The predetermined search procedure yielded a total of 2969 items. The process of selecting the 13 studies for inclusion in the meta-analysis and the method used to exclude studies that did not meet the inclusion criteria are shown in the flowchart shown in Figure 1. After reading the titles and/or abstracts, we eliminated any studies that did not directly address our research question or objectives, as well as any studies that were too small to publish in full. We also disqualified any studies that dealt with HLA in patients with celiac disease in conjunction with another disease. Moreover, *n* = 22 articles were subsequently obtained, and after a review of the full text, the references among these 22 studies were screened, and *n* = 7 studies that deal with CD in children were excluded, as well as two further studies.

We extracted 30,183 patients’ information from 13 studies. Independent references from 13 studies were included in this study. The largest sample size for the individual study was n = 25,025, and n = 49 was the smallest sample size. The other characteristics of this study are summarized in Table 2. Table 2 shows that the majority of CD patients included in the study were female (63%), with a mean age of [42.70 ± 14.21]. The clinical characteristics (Table 3) of the patients included anemia (70%), diarrhea (22%), and autoimmune enteropathy (14%). The histological distribution (Table 4) of CD patients was 70% for Marsh I, 15% for Marsh II, and 14% for Marsh III.

**Figure 2:**
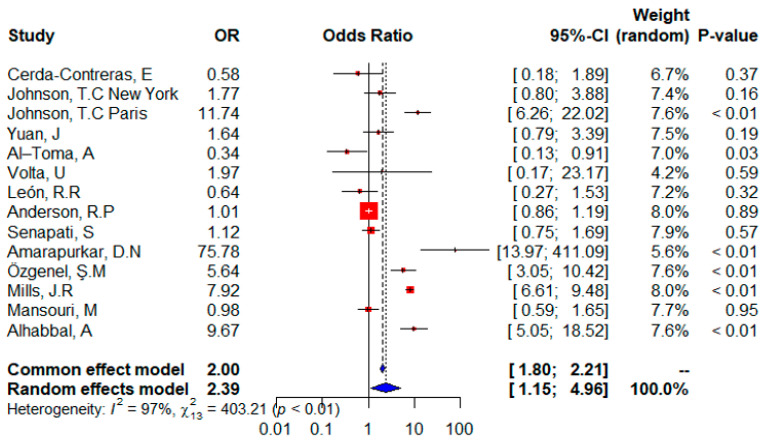
Forest plot of CD risk analysis in adult patients with the HLADQ2 allele. Abbreviations: CI, confidence interval. Data sources are shown in Table 2. In the forest plot the contribution of each study to the meta-analysis (its weight) is represented by the area of a box whose centre represents the size of the odds ratio (OR) estimated from that study (point estimate). The 95% confidence interval (CI) for the OR from each study is also shown. The summary OR is shown by the middle of a diamond whose left and right extremes represent the corresponding CI.

The forest plot (Figure 2) shows that all 13 studies were included in the meta-analysis and provided information on the distribution of the HLA DQ2 gene. The random effects of the meta-analysis show that patients with celiac disease have a higher risk of expressing the HLA DQ2 gene (OR = 2.39; 95% CI, 1.15–4.96), or the risk was significantly higher for a patient with CD (*p*-value = 0.0190) according to the random effects model. Although data from eight isolated studies suggest that HLADQ2 is a significant risk factor, three studies showed that it is not significant, and two studies are neutral for CD patients and report that HLADQ2 has no effect on CD. The overall effect was significant (*p* = 0.0190 by random-effects model *p*-value < 0.0001 by fixed-effects model) and indicated a higher risk of expressing HLADQ2 in a patient with celiac disease. Of note, the heterogeneity (I^2^ = 97%) and heterogeneity tests were significant (𝜒^2^ = 403.21; *p* < 0.01).

**Figure 3:**
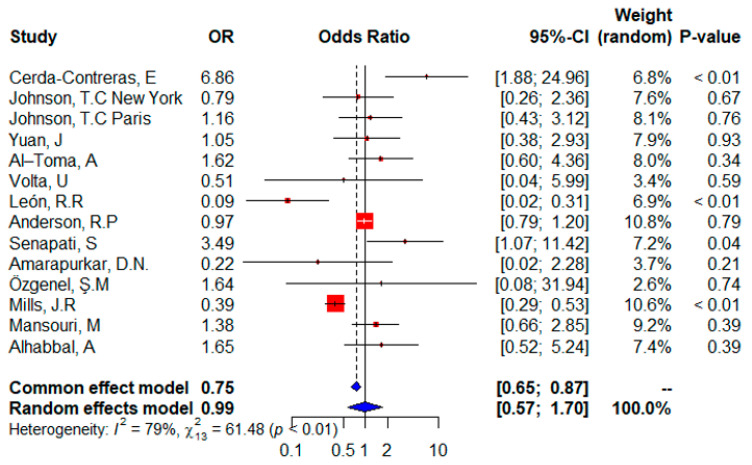
Forest plot of CD risk analysis in adult patients with the HLADQ8 allele. Abbreviations: CI, confidence interval. Data sources are shown in Table 2. In the forest plot the contribution of each study to the meta-analysis (its weight) is represented by the area of a box whose center represents the size of the odds ratio (OR) estimated from that study (point estimate). The 95% confidence interval (CI) for the OR from each study is also shown. The summary OR is shown by the middle of a diamond whose left and right extremes represent the corresponding CI.

The forest plot (Figure 3) shows that all 13 studies were included in the meta-analysis and provided information on the distribution of the HLA DQ8 gene. The random effects of the meta-analysis showed that controls had a higher risk of expressing the HLA DQ8 gene (OR = 0.99, 95% CI, 0.57–1.70); the risk was not statistically significant (*p*-value of 0.9654 according to the random effects model). Although data from eight individual studies suggested that HLADQ8 was a risk factor, five studies showed that it was not significant. The overall effect was significant (*p* = 0.0001) according to the fixed-effect model and indicated a higher risk of expressing the HLADQ8 gene in controls. In addition, heterogeneity was significant (I^2^ = 79%) and the test for heterogeneity was significant (𝜒^2^ = 61.48; *p* < 0.01).

**Figure 4:**
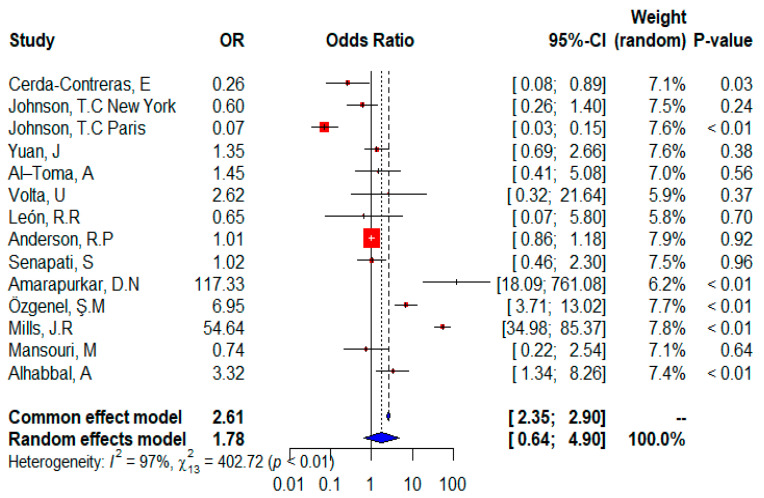
Forest plot of CD risk analysis in adult patients with HLADQ2/DQ8 allele. Abbreviations: CI, confidence interval. Data sources are shown in Table 2. In the forest plot the contribution of each study to the meta-analysis (its weight) is represented by the area of a box whose center represents the size of the odds ratio (OR) estimated from that study (point estimate). The 95% confidence interval (CI) for the OR from each study is also shown. The summary OR is shown by the middle of a diamond whose left and right extremes represent the corresponding CI.

The forest plot (Figure 4) shows that all 13 studies were included in the meta-analysis and provided information on the distribution of the HLADQ2/HLADQ8 gene. The random effects of the meta-analysis showed that patients with celiac disease have a higher risk of expressing the HLA DQ2/DQ8 gene (OR = 1.78, 95% CI, 0.64–4.90), but this risk was not statistically significant (*p* = 0.2663 according to the random-effect model). Although data from seven unique studies suggest that HLADQ2/HLADQ8 is a significant risk factor, five studies showed that it is not significant, and only one study is neutral for CD patients and states that HLADQ2/HLA DQ8 has no effect on CD. The overall effect is significant (*p* < 0.0001 according to the fixed-effect model), indicating a higher risk of expressing HLADQ2/HLADQ8 in CD patients. In addition, considerable heterogeneity (I^2^ = 97%) and test for heterogeneity were significant (𝜒^2^ = 402.72; *p* < 0.01).

**Figure 5:**
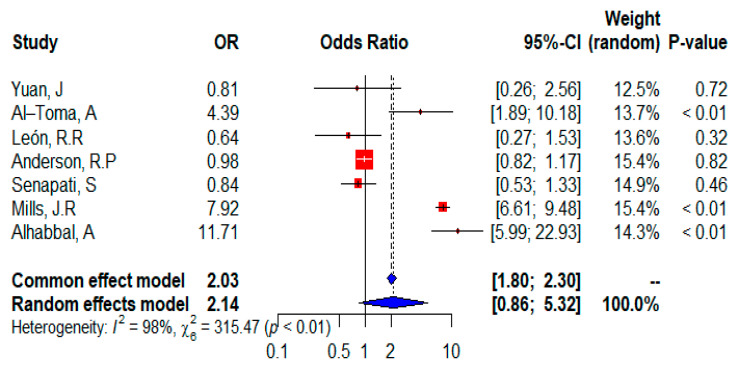
Forest plot of CD risk analysis in adult patients with the homozygous HLADQ2 allele. Abbreviations: CI, confidence interval. Data sources are shown in Table 2. In the forest plot the contribution of each study to the meta-analysis (its weight) is represented by the area of a box whose center represents the size of the odds ratio (OR) estimated from that study (point estimate). The 95% confidence interval (CI) for the OR from each study is also shown. The summary OR is shown by the middle of a diamond whose left and right extremes represent the corresponding CI.

The forest plot (Figure 5) shows that all seven studies were included in the meta-analysis and provided information on the distribution of the homozygous HLA DQ2 allele. The random effects of the meta-analysis showed that patients with celiac disease have a higher risk of expressing the homozygous HLA DQ2 allele (OR = 2.14, 95% CI, 0.86–5.32), or the risk was statistically significant (*p* = 0.1026 according to the random-effect model). Although data from three isolated studies suggest that the homozygous HLAQ2 allele is a significant risk factor, three studies showed that it is not significant, and only one study is neutral for patients with CD and states that the homozygous HLA DQ2 allele has no effect on CD. The overall effect is significant (*p* < 0.0001 according to the fixed-effect model), indicating a higher risk of homozygous HLADQ2 expression in CD patients. In addition, heterogeneity was considerable (I^2^ = 98%) and the test for heterogeneity was significant (𝜒^2^ = 315.47; *p* < 0.01).

**Figure 6:**
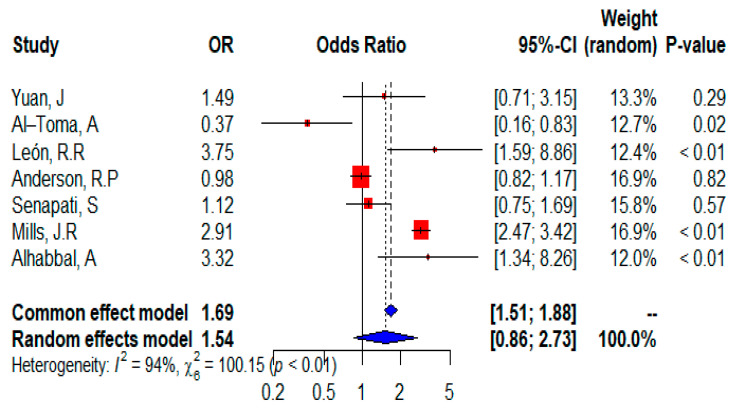
Forest plot of CD risk analysis in adult patients with heterozygous HLADQ2 allele. Abbreviations: CI, confidence interval. Data sources are shown in Table 2. In the forest plot the contribution of each study to the meta-analysis (its weight) is represented by the area of a box whose center represents the size of the odds ratio (OR) estimated from that study (point estimate). The 95% confidence interval (CI) for the OR from each study is also shown. The summary OR is shown by the middle of a diamond whose left and right extremes represent the corresponding CI.

The forest plot (Figure 6) shows that all seven studies were included in the meta-analysis and provided information on the distribution of the heterozygous HLA DQ2 allele. The randomized site effects of the meta-analysis showed that celiac disease patients have a risk of expressing the heterozygous HLA DQ2 allele (OR = 1.54; 95% CI, 0.86–2.73) or the risk was not statistically significant (*p* = 0.1440 in the random-effects model), but in the fixed-effects model (*p* < 0.0001). Although data from five isolated studies suggest that the heterozygous HLAQ2 allele is a significant risk factor, one study showed that it is not significant, and only one study is neutral for CD patients and reports that the heterozygous HLA DQ2 allele has no effect on CD. In addition, heterogeneity was considerable (I^2^ = 94%) and the test for heterogeneity was significant (𝜒^2^ = 100.15; *p* < 0.01).

**Figure 7:**
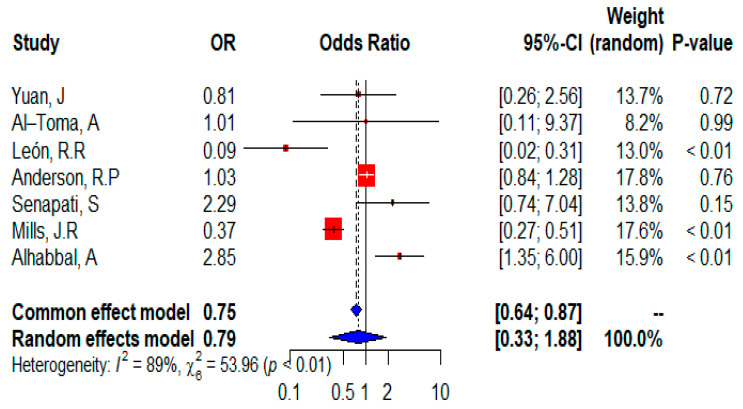
Forest plot of CD risk analysis in adult patients with heterozygous HLADQ8 allele. Abbreviations: CI, confidence interval. Data sources are shown in Table 2. In the forest plot the contribution of each study to the meta-analysis (its weight) is represented by the area of a box whose center represents the size of the odds ratio (OR) estimated from that study (point estimate). The 95% confidence interval (CI) for the OR from each study is also shown. The summary OR is shown by the middle of a diamond whose left and right extremes represent the corresponding CI.

The forest plot (Figure 7) shows that all seven studies were included in the meta-analysis and provided information on the distribution of the heterozygous HLA DQ8 allele. The random effects of the meta-analysis showed that controls had a higher risk of expressing the heterozygous HLA DQ8 allele (OR = 0.79 95% CI, 0.33–1.88) or the risk was not statistically significant (*p* = 0.5999 according to the random-effects model). However, according to the fixed-effects model (*p* = 0.0002). Although data from two single studies suggest that the heterozygous HLAQ8 allele is a significant risk factor, three studies showed that it is not significant, and two studies are neutral for CD patients and report that the heterozygous HLA DQ8 allele has no effect on CD. Heterogeneity was significant (I^2^ = 89%) and the test for heterogeneity was significant (𝜒^2^ = 53.96; *p* < 0.01).

## 5. Discussion

This research is the first comprehensive meta-analysis of the DQ2 and DQ8 heterodimers predisposing to CD that are encoded by the HLA-DQB1 and HLA-DQA1 alleles in adults with CD. Indeed, it provided 3158 adult CD patients and 26,863 controls for statistical analysis. Numerous commercial and university labs provide HLA genetic testing, which is used to diagnose celiac disease and find people at risk in the same families. Celiac disease HLA testing has a poor positive predictive value but a high negative predictive value. As a result, it is crucial for the practicing physician to know when to ask for an HLA genetic test [33]. In symptomatic individuals who have already begun a gluten-free diet, HLA testing may be used to practically rule out celiac disease [34]. This has been shown by several researchers [35]. Further clarification of a diagnosis may be obtained by HLA testing. HLA testing, for instance, may be used to rule out celiac disease if HLA-DQ2 or DQ8 are missing and would require additional testing if DQ2 or DQ8 are discovered in those with inconclusive serological or biopsy findings and/or inadequate gluten elimination [34,36].

Recent data on microbiome studies in CD show the importance of broader HLA screening, as some bacteria have been associated with CD in the absence of classical HLA risk alleles. Knowledge of the degree of relevance of classical HLA associations could be included in risk assessment algorithms in different populations, also taking into account a wider range of HLA haplotypes [37]. We introduced some clinical features of the patients, such as diarrhea, anemia, and autoimmune enteropathy. Of these, we found that anemia constituted the highest percentage (35%) in adult CD patients. Indeed a study in Danish adult CD patients also found (30%) of them suffered from anemia, which could be the main symptom of CD [38]. On the other hand, 23% of patients had anemia at diagnosis, as reported by Saukkonen et al. [39].

We found that diarrhea constitutes a percentage (22%) in adult patients with CD. Among these, we found that diarrhea constitutes the highest percentage (18.6%) in adult CD patients. Some previous studies [9,40] have confirmed that diarrhea is the most common symptom in adult CD patients [40]. However, the presence of extra-intestinal symptoms and atypical clinical forms is very common and explains the many undiagnosed cases. In this view, the identification of HLA risk haplotypes in populations could be one of the main issues to be considered for the prediction of celiac risk and to exclude CD diagnosis in individuals. Autoimmune enteropathy was present in 14% of patients in this study. Moreover, a previous study showed that it constitutes a rare cause of refractory diarrhea in the presence of autoantibodies in the serum and enteropathy of the small intestine [41,42]. The histological distribution (Table 4) of CD patients was 70% for Marsh I, 15% for Marsh II, and 14% for Marsh III. The modified Marsh–Oberhuber classification can be used to classify the histological features associated with CD [43]. Our results indicate that the majority of adult patients present Marsh I; however, a recent study performed on a Chinese adult population showed that Among 69 CD patients, nine had Marsh grade I and 50 had Marsh grade ≥2. The histological findings of CD included total villous atrophy, increased intraepithelial lymphocytes, and cryptic hyperplasia [44]. 

We observed that the risk of getting CD in individuals with DQ2, which is encoded by the HLA-DQA1*05 and DQB1*02 alleles, has been generally verified. This was the conclusion we came to after doing our research. According to the findings of this study, the odds ratio for the double dose of HLA-DQB1*02 in adult CD patients was significantly higher than in controls (OR = 2.39; *p* = 0.0190 according to the random effect model; *p* < 0.0001 according to the fixed effect model), and the odds ratio for the single dose of HLA-DQB1*02 in adult CD patients was also relatively increased in comparison to controls (OR = 2.14) However, these results were not significant according to *p* = 0.1440 of the random effect model, the fixed effect model shows a significant *p* = 0.0001. This result is similar to that [45]. It is shown that having a twofold dosage of the HLA-DQB1*02 allele was linked to an odds ratio of more than five for the development of juvenile CD, independent of the presence of other HLA-DQ alleles. Additionally, even a single “dose” of HLA-DQB1*02 was linked with a rather significant chance of developing the disease (OR around 4). The HLA-DQA1 and HLA-DQB1 genes, which are responsible for encoding HLA-DQ2 and HLA-DQ8, are the primary genetic factors that determine a person’s susceptibility to disease [46] and were found in 69.5% of Iranian CD patients [47]. In Brazil, HLA DQ2 was present in 75.6% of CD patients [5]. The proportion of CD patients who are likely to express the heterodimer DQ (A1*0501, B1*0201) was 91% in the United Kingdom, 87% in Rome, Italy, and 82% in Bologna, Italy [48]. A significant frequency of HLA-DQ2/DQ8 alleles was also found in the FDR of CD patients, according to prior research [49].

In a cross-sectional examination conducted in Brazil, HLA-DQ2/DQ8 was found in 98.4% of people with celiac disease, 89.6% of those with celiac disease in their families, and 55.4% of people in the general population who did not have familial celiac disease [50]. Recently, Mansouri et al. showed that HLA-DQ2/DQ8 patients represent a rate was 73.4% [31].

The HLA-DQ2 variation was shown to be more prevalent in CD patients in the Arab community. Indeed, the DQB1*02 allele was present in 84.6% of Palestinians, 77.42% of Egyptians, and 45.2% of Moroccans who had celiac disease. Furthermore, 48.0% of the anti-TTG-positive group in Libya was homozygous for the HLA-DQ2 gene, and the same was true for the Kingdom of Saudi Arabia. 52.7% of individuals tested positive for HLA-DQ molecules that are linked with CD DQ2 or DQ8 [47,51,52,53,54].

The study of the homozygous DQB1*02 is important because the double dose of this allele is linked to the early development of this pathology as well as to an increase in anti-TTG levels, severity, and complications [55,56,57].

Differences between studies found in this meta-analysis could be due to differences in the frequencies of HLA haplotypes in the analyzed populations. Studies on the frequency of different genetic loci and anthropological traits clearly illustrate the geographical and genetic barrier between Europeans and Asians across northwest China. Among the many Chinese communities, the northwestern population is more closely linked to Europeans than the other populations in southern China [58,59].

This significant heterogeneity in gene frequencies that has been found by studies may also be due to the different numbers of participants and the variance in accuracy associated with the different methods used for HLA class II typing (such as PCR-SSO, PCR-RFLP, PCR-SSP, and PCR-SBT) [60]. 

In adult patients with CD, the frequency of HLA DQ8 is lower than in controls (OR = 0.79) *p* = 0.5999 according to the random effect model is not statistically significant, but according to the fixed effect, the *p*-value is significant (*p* = 0.0002). These results were in agreement with the lower risk associated with the DQ8 variant previously described in Moroccan and Libyan patients due to the higher frequency of DQ8 in controls [54].

In the European population, the frequency of this variant is 17.8% in CD patients [20]. The frequency of the HLA DQ2 variant is 90%, while the frequency of HLA DQ8 is between 5% and 10% [57]. HLA-DQ8 haplotype (10.2%) is among the first-degree relatives (FDRs) with the heterozygous HLADQ8 haplotype 9.4% were heterozygous HLA-DQ8. However, only one FDR was homozygous HLA-DQ8 (0.08%) [61].

The condition is very infrequent in those who do not possess HLA-DQ alleles, according to the research of other scientists [5]. CD is related to HLA DQ8 (DQA1*0301 and DQB1*0302) to a lesser extent [62]. The incidence of celiac patients in Europe who do not express HLA DQ8 or HLA DQ8 is less than 0.5% because CD patients carry at least half the DQ2.10 haplotype [63,64,65,66]. In addition, 33.33% of individuals who did not display DQ2 but were positive for DQ8 were found to be homozygous for DQ8 (DQA1*03- DQB1*0302). 18% of patients who did not have the DQ2 gene but did have the DQ8 gene were carriers of the DRB1*07-DQB1*02 haplotype [5]. 

In our analysis, heterozygous DQ8 did not increase the risk of CD. An Arab population case-control research supported these findings, showing that the frequency of heterozygous HLADQ8 was identical in cases and controls, and so represented a risk for the general population (1:70–79 versus 1:67 in the general population) [67].

In our analysis, heterozygous DQ8 did not increase the risk of CD; this result corroborates the previous observation that carriers of the heterozygous DQ8 genotype (2.11%) have lower EMA than homozygous DQ8 celiac individuals (8.42%) [68]. The HLA DQ2 and/or HLADQ8 status in adult celiac patients was higher than that of controls. Indeed, our findings are in line with those of other Brazilian studies that were conducted in the past; the DQ2 and DQ8 genotypes were found in 93.2% of celiac patients in the northeastern region of the country [69]. These results were similar to those performed by Cecilio and Bonatto [50], who hypothesized that individuals with celiac disease had a frequency of HLA DQ2/DQ8 of 98.4% and 89.6% of parents of celiac patients [50]. As in another study in Spain, celiac patients positive for the HLADQ2 /HLADQ8 variants were 98% compared to 49.1% of non-diseased controls (*p* < 0.001, OR: 51.57) [70].

In Figure 2 and Figure 4, the two studies from India agree with the direction (although their significance is different), whereas in Figure 3, the two studies have different effects, indeed recently, in the general indigenous population of South India, the prevalence of HLA-DQ8 is higher than the prevalence of HLA-DQ2. This finding could be related to the late introduction of wheat into the diet of the South Indian population [71].

The European population showed differences in effect and significance, and this difference could be due to combinations of population-related genetic and environmental factors. Many questions are still open regarding the role and interactions between genetic and environmental factors in the development of CD. Environmental factors are mainly associated with the introduction of gluten (timing, amount, breastfeeding, etc.) [54]. Asian population showed similar effects in effect and significance (Figure 2, Figure 3 and Figure 4), and CD is emerging in many Asian countries. In addition, the Asia Pacific Association for Gastroenterology has established a formal working group on celiac disease to conduct relevant research to reduce the burden of DAC in Asia [72], and the American population showed the same effect and significance (Figure 2 and Figure 3) which is different to that of North America. This could be due to the environmental difference between North and South America.

Full HLA-DQ genotyping might be kept for adults with clinical suspicion (the most prevalent symptoms in adult CD patients are diarrhea and autoimmune enteropathy; histological results have shown that the majority of adult CD patients have Marsh I in their intestinal mucosa). However, many of the undetected cases might be attributed to extra-intestinal symptoms of unusual clinical presentations. Recent work by Verma et al. presented a quick HLA-DQ typing approach for identifying CD patients who have susceptibility genes. The researchers essentially ran polymerase chain reaction (PCR) on blood samples from CD patients, FDRs, and controls using a kit containing primers for HLA-DQ target alleles exclusively. In terms of the presence or absence of HLA-DQ2 and HLA-DQ8 alleles, they could exhibit good agreement with the findings obtained by traditional high-resolution HLA-DQ typing [73]. Although CD has been widely researched and characterized in the West, it is still difficult to make an evidence-based diagnosis in many developing nations due to obstacles in the health care system [10,74,75]. Determining the genetic risk of celiac disease early in life may allow subsequent antibody monitoring in older adults [76].

## 6. Conclusions

The results obtained confirmed that the DQ2 allele is the primary one related to CD due to its high frequency in adult patients in all studies; the homozygous and heterozygous status of HLADQ2 is present with increased frequency in most adult patients. The high immunodominance and the pathogenic mechanisms of gluten peptide presentation by DQ2 contribute to the close connection between HLA haplotypes and celiac disease. Although the classical DQ2/DQ8 associations with CD were confirmed in this meta-analysis, a minority of CD cases develop in the absence of predisposing HLA haplotypes. A detailed analysis of MHC in DQ2-negative CD should lead to a better understanding of the susceptibility genes for CD. Recent data on microbiome studies in CD [37] evidence the importance of a broader HLA screening, as some bacteria were associated with CD in the absence of the classical HLA risk alleles. At present, the real contribution of non-HLA genes is still unknown. In the future, the knowledge of the grade of the relevance of HLA associations could be included in algorithms for the evaluation of the risk in different populations, also taking into account a broader range of HLA haplotypes. These observations may contribute to the debate on the potential and cost-effective implementation of broader or mass screening strategies for CD in adults. In addition, the determination of genetic risk for celiac disease early in life may allow subsequent antibody monitoring in older adults (Ministry of Health).

## Figures and Tables

**Table 1:** Results of quality evaluation using QUADAS.

	[20]	[21]	[21]	[22]	[23]	[24]	[25]	[26]	[27]	[28]	[29]	[30]	[31]	[32]
*1. Was the spectrum of patients representative of the patients who will receive the test in practice?*	N	Y		Y	Y	Y	Y	*Y*	*Y*	Y	Y	Y	Y	Y
*2-Were selection criteria clearly described?*	Y	Y	Y	Y	Y	*Y*	Y	Y	Y	Y	Y	Y	Y	Y
*3-Is the reference standard likely to correctly classify the target condition?*	U	U	U	U	U	U	Y	Y	*U*	Y	Y	Y	Y	Y
*4-Is the time period between the reference standard and index test short enough to be reasonably sure that the target condition did not change between the two tests?*	U	Y	Y	Y	U	U	U	Y	Y	U	U	U	Y	Y
*5-sure that the target condition did not change between the two tests?*	Y	U	U	Y	Y	Y	Y	*Y*	Y	Y	U	Y	Y	Y
*6-Did the whole sample or a random selection of the sample receive verification using a reference standard of diagnosis?*	Y	Y	Y	Y	Y	Y	Y	Y	Y	Y	Y	Y	Y	Y
*7-Was the reference standard independent of the index test (i.e., the index test did not form part of the reference standard)?*	Y	Y	Y	Y	Y	Y	Y	Y	Y	Y	Y	Y	Y	*Y*
*8-Was the execution of the index test described in sufficient detail to permit replication of the test?*	Y	Y	Y	Y	Y	Y	Y	Y	Y	Y	Y	Y	Y	Y
*9-Was the execution of the reference standard described in sufficient detail to permit its replication?*	U	U	U	U	U	U	U	U	U	U	U	U	U	U
*10-Were the index test results interpreted without knowledge of the results of the reference standard?*	Y	Y	Y	U	U	Y	Y	U	Y	Y	Y	Y	Y	Y
*11-Were the reference standard results interpreted without knowledge of the results of the index test?*	Y	Y	Y	Y	Y	*Y*	Y	Y	Y	Y	Y	Y	Y	Y
*12-Were the same clinical data available when test results were interpreted as would be available when the test is used in practice?*	Y	Y	Y	Y	Y	Y	Y	Y	Y	Y	Y	Y	Y	Y
*13-Were uninterpretable/intermediate test results reported?*	*Y*	*Y*	*Y*	*Y*	*Y*	*Y*	Y	*Y*	*Y*	*Y*	*Y*	Y	Y	Y
*14-Were withdrawals from the study explained?*	Y	Y	Y	Y	Y	Y	Y	Y	Y	Y	Y	Y	Y	Y

Y: Yes, N: No, U: Unclear.

**Table 2:** Characteristics of Included Studies on CD Patients.

Author	Years of Publication	Country (City)	Number of Participants	Patient/ Controls	Gender Men/ Women	Age at Diagnosis (Years)	Study Type	Diagnosis
[20]	2018	Mexico, (Mexico)	49	30/19	11/38	53.0 ± 14.5	Exploratory study	Serology: IgA/IgG anti-tTG, and IgA EmA Endoscopy HLA
[21]	2004	USA, (New York)	104	44/60	18/26	46.2	Cohort study	Histology HLA typing
[21]	2004	France, (Paris)	507	66/441	14/12	21.7	Cohort study	Histology HLA typing
[22]	2017	China, (Jiangxi)	147	59/88	-	18.8 ± 1.1	Cross-sectional prospective study	Serology IgG anti-DGP IgA anti-tTG HLA typing
[23]	2006	Nerthlands, (Arnhem)	151	30/121	16/14	61.5 [52–79]	-	Histology Endoscopy Serology IgA, IgG, and IgA EmA
[24]	2015	Italy, (Bologna)	77	61/16	15/46	36 [14–66]	Prospective cohort study	HLA typing Histology
[25]	2019	Spain, (Madrid)	223	197/26	76/213	15–89	Retrospective single-center study	Histology Serology Anti-Tg2 and/or EMA antibodies IgG (DGP IgG) and/or anti-tissue transglutaminase 2 IgG (antiTg2 IgG) Endoscopy HLA typing
[26]	2013	Australia, (Parkville)	2548	1390/1158	1.158 /1.390	20–97	Cohort study	Histology Serology HLA typing
[28]	2016	India, (Delhi)	557	340/217	345/526	35.34 ± 11.83	Cohort study	Serology Anti-TTG Illumina microarray genotyping.
[27]	2015	India, (Mumbai)	59	34/25	8/26	38.79 ± 15.84	Retrospective study	Serology Histology HLA typing
[29]	2019	Turkey, (Eskisehir)	285	94/102	30/64	>18 years	-	Serology IgA, IgG transglutaminase Anti tTG, Anti EMA antibodies Antigliadin Histology
[30]	2020	USA, (Rochester)	25,025	613/24,339	185/428	17.6 years	Cohort study	Serology Anti-IgA, Anti-tTG Endoscopy Histology
[31]	2021	Iran, (Tehran)	251	100/151	49/51	30–35	Case-control study	Serology Anti tTG HLA typing
[32]	2021	Syria, (Damasens)	200	100/100	51/49	22 ± 14	Case control study	Serology Anti tTG/EMA Histology

**Table 3:** Clinical characterizations of population.

Author	Number of Patients CD	Age at Diagnosis, Years	Diarrhea	Autoimmune Enteropathy	Anemia
[20]	30	54.2	1	2	0
[21]	44	46.2	20	0	0
[21]	66	21.7	59	0	0
[22]	59	20.5	7	13	0
[23]	30	61.5	0	0	0
[24]	61	40	10	22	
[25]	223	52	86	84	86
[26]	2548	58.5	2	0	0
[28]	557	35.34	606	6	167
[27]	59	38.79	20	0	26
[31]	40	22	6	0	25

0 not available.

**Table 4:** Histological characteristics of patients with CD.

Author	Marsh I	Marsh II	Marsh III
[23]	43	43	30
[24]	46	0	0
[25]	11	2	276
[28]	92	139	640
[27]	31	10	11
[32]	3	11	58

0 not available.

## Data Availability

Not applicable.

## References

[1] Fasano A., Catassi C. (2001). Current Approaches to Diagnosis and Treatment of Celiac Disease: An Evolving Spectrum. Gastroenterology.

[2] Green P.H., Cellier C. (2007). Celiac Disease. N. Engl. J. Med..

[3] Kuja-Halkola R., Lebwohl B., Halfvarson J., Wijmenga C., Magnusson P.K., Ludvigsson J.F. (2016). Heritability of Non-HLA Genetics in Coeliac Disease: A Population-Based Study in 107,000 Twins. Gut.

[4] Wolters V.M., Wijmenga C. (2008). Genetic Background of Celiac Disease and Its Clinical Implications. Off. J. Am. Coll. Gastroenterol. ACG.

[5] Karell K., Louka A.S., Moodie S.J., Ascher H., Clot F., Greco L., Ciclitira P.J., Sollid L.M., Partanen J. (2003). HLA Types in Celiac Disease Patients Not Carrying the DQA1* 05-DQB1* 02 (DQ2) Heterodimer: Results from the European Genetics Cluster on Celiac Disease. Hum. Immunol..

[6] Fasano A., Catassi C. (2012). Celiac Disease. N. Engl. J. Med..

[7] Singh P., Arora S., Singh A., Strand T.A., Makharia G.K. (2016). Prevalence of Celiac Disease in Asia: A Systematic Review and Meta-Analysis. J. Gastroenterol. Hepatol..

[8] Catassi C., Gatti S., Fasano A. (2014). The New Epidemiology of Celiac Disease. J. Pediatr. Gastroenterol. Nutr..

[9] Rubio-Tapia A., Ludvigsson J.F., Brantner T.L., Murray J.A., Everhart J.E. (2012). The Prevalence of Celiac Disease in the United States. Off. J. Am. Coll. Gastroenterol. ACG.

[10] Singh P., Arora A., Strand T.A., Leffler D.A., Catassi C., Green P.H., Kelly C.P., Ahuja V., Makharia G.K. (2018). Global Prevalence of Celiac Disease: Systematic Review and Meta-Analysis. Clin. Gastroenterol. Hepatol..

[11] Ashtari S., Najafimehr H., Pourhoseingholi M.A., Rostami K., Asadzadeh-Aghdaei H., Rostami-Nejad M., Tavirani M.R., Olfatifar M., Makharia G.K., Zali M.R. (2021). Prevalence of Celiac Disease in Low and High Risk Population in Asia–Pacific Region: A Systematic Review and Meta-Analysis. Sci. Rep..

[12] Lionetti E., Catassi C. (2014). Co-Localization of Gluten Consumption and HLA-DQ2 and-DQ8 Genotypes, a Clue to the History of Celiac Disease. Dig. Liver Dis..

[13] Duerksen D.R., Wilhelm-Boyles C., Parry D.M. (2005). Intestinal Permeability in Long-Term Follow-up of Patients with Celiac Disease on a Gluten-Free Diet. Dig. Dis. Sci..

[14] Hollon J., Puppa E.L., Greenwald B., Goldberg E., Guerrerio A., Fasano A. (2015). Effect of Gliadin on Permeability of Intestinal Biopsy Explants from Celiac Disease Patients and Patients with Non-Celiac Gluten Sensitivity. Nutrients.

[15] Bledsoe A.C., King K.S., Larson J.J., Snyder M., Absah I., Murray J.A. (2019). Micronutrient Deficiencies Are Common in Contemporary Celiac Disease despite Lack of Overt Malabsorption Symptoms. Mayo Clin. Proc..

[16] Corrao G., Corazza G.R., Bagnardi V., Brusco G., Ciacci C., Cottone M., Guidetti C.S., Usai P., Cesari P., Pelli M.A. (2001). Mortality in Patients with Coeliac Disease and Their Relatives: A Cohort Study. Lancet.

[17] Lepers S., Couignoux S., Colombel J.-F., Dubucquoi S. (2004). La Maladie Cøeliaque de l’adulte: Aspects Nouveaux. Rev. Méd. Intern..

[18] Roszkowska A., Pawlicka M., Mroczek A., Balabuszek K., Nieradko-Iwanicka B. (2019). Non-Celiac Gluten Sensitivity: A Review. Medicina.

[19] Whiting P., Rutjes A.W., Reitsma J.B., Bossuyt P.M., Kleijnen J. (2003). The Development of QUADAS: A Tool for the Quality Assessment of Studies of Diagnostic Accuracy Included in Systematic Reviews. BMC Med. Res. Methodol..

[20] Cerda-Contreras E., Ramírez-Cervantes K.L., Granados J., Mena L., Núñez-Álvarez C., Uscanga L. (2018). Is Celiac Disease Better Identified through HLA-DQ8 than through HLA-DQ2 in Mexican Subjects?. Rev. Gastroenterol. México.

[21] Johnson T.C., Diamond B., Memeo L., Negulescu H., Hovhanissyan Z., Verkarre V., Rotterdam H., Fasano A., Caillat-Zucman S., Grosdidier E. (2004). Relationship of HLA-DQ8 and Severity of Celiac Disease: Comparison of New York and Parisian Cohorts. Clin. Gastroenterol. Hepatol..

[22] Yuan J., Zhou C., Gao J., Li J., Yu F., Lu J., Li X., Wang X., Tong P., Wu Z. (2017). Prevalence of Celiac Disease Autoimmunity among Adolescents and Young Adults in China. Clin. Gastroenterol. Hepatol..

[23] Al–Toma A., Goerres M.S., Meijer J.W., Peña A.S., Crusius J.B.A., Mulder C.J. (2006). Human Leukocyte Antigen–DQ2 Homozygosity and the Development of Refractory Celiac Disease and Enteropathy-Associated T-Cell Lymphoma. Clin. Gastroenterol. Hepatol..

[24] Volta U., Caio G., Giancola F., Rhoden K.J., Ruggeri E., Boschetti E., Stanghellini V., De Giorgio R. (2016). Features and Progression of Potential Celiac Disease in Adults. Clin. Gastroenterol. Hepatol..

[25] León R.R., Pérez L.C., de Santiago E.R., Ariño G.R., Martín A.D.A., Jiménez C.G.H., Rodríguez E.S., González A.S., Prieto F.L., Albillos A. (2019). Genetic and Flow Cytometry Analysis of Seronegative Celiac Disease: A Cohort Study. Scand. J. Gastroenterol..

[26] Anderson R.P., Henry M.J., Taylor R., Duncan E.L., Danoy P., Costa M.J., Addison K., Tye-Din J.A., Kotowicz M.A., Knight R.E. (2013). A Novel Serogenetic Approach Determines the Community Prevalence of Celiac Disease and Informs Improved Diagnostic Pathways. BMC Med..

[27] Senapati S., Sood A., Midha V., Sood N., Sharma S., Kumar L., Thelma B.K. (2016). Shared and Unique Common Genetic Determinants between Pediatric and Adult Celiac Disease. BMC Med. Genom..

[28] Amarapurkar D.N., Somani V.S., Shah A.S., Kankonkar S.R. (2016). HLA–DQ Genotyping in Celiac Disease in Western India. Trop. Gastroenterol..

[29] Özgenel Ş.M., Temel T., Teke H.Ü., Yıldız P., Korkmaz H., Özakyol A. (2019). HLA-DQ2/DQ8 Frequency in Adult Patients with Celiac Disease, Their First-Degree Relatives, and Normal Population in Turkey. Turk. J. Gastroenterol..

[30] Choung R.S., Mills J.R., Snyder M.R., Murray J.A., Gandhi M.J. (2020). Celiac Disease Risk Stratification Based on HLA-DQ Heterodimer (HLA-DQA1~DQB1) Typing in a Large Cohort of Adults with Suspected Celiac Disease. Hum. Immunol..

[31] Mansouri M., Dadfar M., Rostami-Nejad M., Ekhlasi G., Shahbazkhani A., Shahbazkhani B. (2021). The Frequency of HLA-DQ2/DQ8 Haplotypes and Celiac Disease among the First-Degree Relatives of Patients with Celiac Disease. Gastroenterol. Hepatol. Bed Bench.

[32] Alhabbal A., Abou Khamis I. (2021). HLA-DQ Genotyping of Celiac Disease among Syrian Patients. Ann. Rom. Soc. Cell Biol..

[33] Brown N.K., Guandalini S., Semrad C., Kupfer S.S. (2019). A Clinician’s Guide to Celiac Disease HLA Genetics. Off. J. Am. Coll. Gastroenterol. ACG.

[34] Kaukinen K., Partanen J., Mäki M., Collin P. (2002). HLA-DQ Typing in the Diagnosis of Celiac Disease. Am. J. Gastroenterol..

[35] Rostom A., Murray J.A., Kagnoff M.F. (2006). American Gastroenterological Association (AGA) Institute Technical Review on the Diagnosis and Management of Celiac Disease. Gastroenterology.

[36] Rubio-Tapia A., Hill I.D., Kelly C.P., Calderwood A.H., Murray J.A. (2013). ACG Clinical Guidelines: Diagnosis and Management of Celiac Disease. Off. J. Am. Coll. Gastroenterol. ACG.

[37] Milletich P.L., Ahrens A.P., Russell J.T., Petrone J.R., Berryman M.A., Agardh D., Ludvigsson J.F., Triplett E.W., Ludvigsson J. (2022). Gut Microbiome Markers in Subgroups of HLA Class II Genotyped Infants Signal Future Celiac Disease in the General Population: ABIS Study. Front. Cell. Infect. Microbiol..

[38] Schøsler L., Christensen L.A., Hvas C.L. (2016). Symptoms and Findings in Adult-Onset Celiac Disease in a Historical Danish Patient Cohort. Scand. J. Gastroenterol..

[39] Saukkonen J., Kaukinen K., Koivisto A.-M., Mäki M., Laurila K., Sievänen H., Collin P., Kurppa K. (2017). Clinical Characteristics and the Dietary Response in Celiac Disease Patients Presenting with or without Anemia. J. Clin. Gastroenterol..

[40] Bul V., Sleesman B., Boulay B. (2016). Celiac Disease Presenting as Profound Diarrhea and Weight Loss—A Celiac Crisis. Am. J. Case Rep..

[41] Gentile N.M., Murray J.A., Pardi D.S. (2012). Autoimmune Enteropathy: A Review and Update of Clinical Management. Curr. Gastroenterol. Rep..

[42] Quirós A.B., Sanz E.A., Ordiz D.B., Adrados J.G. (2009). From Autoimmune Enteropathy to the IPEX (Immune Dysfunction, Polyendocrinopathy, Enteropathy, X-Linked) Syndrome. Allergol. Immunopathol..

[43] Kamboj A.K., Oxentenko A.S. (2017). Clinical and Histologic Mimickers of Celiac Disease. Clin. Transl. Gastroenterol..

[44] Wang M., Kong W.-J., Feng Y., Lu J.-J., Hui W.-J., Liu W.-D., Li Z.-Q., Shi T., Cui M., Sun Z.-Z. (2022). Epidemiological, Clinical, and Histological Presentation of Celiac Disease in Northwest China. World J. Gastroenterol..

[45] Capittini C., De Silvestri A., Rebuffi C., Tinelli C., Poddighe D. (2019). Relevance of HLA-DQB1* 02 Allele in the Genetic Predisposition of Children with Celiac Disease: Additional Cues from a Meta-Analysis. Medicina.

[46] Martina S., Fabiola F., Federica G., Chiara B., Gioacchino L., Francesco D.M., Gian L. (2018). de’Angelis Genetic Susceptibilty and Celiac Disease: What Role Do HLA Haplotypes Play?. Acta Bio Med. Atenei Parm..

[47] Mohammed M.A., Omar N.M., Shebl A.M., Mansour A.H., Elmasry E., Othman G. (2014). Celiac Disease Prevalence and Its HLA-Genotypic Profile in Egyptian Patients with Type 1 Diabetes Mellitus. Trends Med. Res..

[48] Hall M.A., Mazzilli M.C., Satz M.L., Barboni F., Bartova A., Brunnler G., Ciclitira P.J., Corazza G.R., Ferrante P., Gerok W. (1991). Coeliac Disease Study. HLA.

[49] Vaquero L., Caminero A., Nuñez A., Hernando M., Iglesias C., Casqueiro J., Vivas S. (2014). Coeliac Disease Screening in First-Degree Relatives on the Basis of Biopsy and Genetic Risk. Eur. J. Gastroenterol. Hepatol..

[50] Cecilio L.A., Bonatto M.W. (2015). The Prevalence of HLA DQ2 and DQ8 in Patients with Celiac Disease, in Family and in General Population. ABCD Arq. Bras. Cir. Dig..

[51] Al-Hussaini A., Alharthi H., Osman A., Eltayeb-Elsheikh N., Chentoufi A. (2018). Genetic Susceptibility for Celiac Disease Is Highly Prevalent in the Saudi Population. Saudi J. Gastroenterol..

[52] Ayesh B.M., Zaqout E.K., Yassin M.M. (2017). HLA-DQ2 and-DQ8 Haplotypes Frequency and Diagnostic Utility in Celiac Disease Patients of Gaza Strip, Palestine. Autoimmun. Highlights.

[53] Ghawil M., Miotti V., Tonutti E., Tenore A., Hadeed I., Sindici C., Visentini D., Morgham A., Abusrewil S. (2012). HLA-DQ Types of Celiac Disease in Libyan Children with Type 1 Diabetes Mellitus. Eur. J. Gastroenterol. Hepatol..

[54] Piancatelli D., Ben El Barhdadi I., Oumhani K., Sebastiani P., Colanardi A., Essaid A. (2017). HLA Typing and Celiac Disease in Moroccans. Med. Sci..

[55] Karinen H., Kärkkäinen P., Pihlajamäki J., Janatuinen E., Heikkinen M., Julkunen R., Kosma V.-M., Naukkarinen A., Laakso M. (2006). Gene Dose Effect of the DQB1* 0201 Allele Contributes to Severity of Coeliac Disease. Scand. J. Gastroenterol..

[56] Nenna R., Mora B., Megiorni F., Mazzilli M.C., Magliocca F.M., Tiberti C., Bonamico M. (2008). HLA-DQB1* 02 Dose Effect on RIA Anti-Tissue Transglutaminase Autoantibody Levels and Clinicopathological Expressivity of Celiac Disease. J. Pediatr. Gastroenterol. Nutr..

[57] Romanos J., Van Diemen C.C., Nolte I.M., Trynka G., Zhernakova A., Fu J., Bardella M.T., Barisani D., McManus R., Van Heel D.A. (2009). Analysis of HLA and Non-HLA Alleles Can Identify Individuals at High Risk for Celiac Disease. Gastroenterology.

[58] Chunjie X., Ruopu D., Cavalli-Sforza L.L. (2000). The Principle Component Analysis of Allele Frequencies of Chinese Groups. Sci. China.

[59] Xue F., Wang J., Hu P., Ma D., Liu J., Li G., Zhang L., Wu M., Sun G., Hou H. (2005). Identification of Spatial Genetic Boundaries Using a Multifractal Model in Human Population Genetics. Hum. Biol..

[60] Yuan J., Gao J., Li X., Liu F., Wijmenga C., Chen H., Gilissen L.J. (2013). The Tip of the “Celiac Iceberg” in China: A Systematic Review and Meta-Analysis. PLoS ONE.

[61] Mishra A., Prakash S., Kaur G., Sreenivas V., Ahuja V., Gupta S.D., Makharia G.K. (2016). Prevalence of Celiac Disease among First-Degree Relatives of Indian Celiac Disease Patients. Dig. Liver Dis..

[62] Tüysüz B., Dursun A., Kutlu T., Sökücü S., Çine N., Süoğlu Ö., Erkan T., Erginel-Ünaltuna N., Tümay G. (2001). HLA-DQ Alleles in Patients with Celiac Disease in Turkey. Tissue Antigens.

[63] Babron M.-C., Nilsson S., Adamovic S., Naluai Å.T., Wahlström J., Ascher H., Ciclitira P.J., Sollid L.M., Partanen J., Greco L. (2003). Meta and Pooled Analysis of European Coeliac Disease Data. Eur. J. Hum. Genet..

[64] Bourgey M., Calcagno G., Tinto N., Gennarelli D., Margaritte-Jeannin P., Greco L., Limongelli M.G., Esposito O., Marano C., Troncone R. (2007). HLA Related Genetic Risk for Coeliac Disease. Gut.

[65] Sollid L.M. (2002). Coeliac Disease: Dissecting a Complex Inflammatory Disorder. Nat. Rev. Immunol..

[66] Wong R.C., Steele R.H., Reeves G.E., Wilson R.J., Pink A., Adelstein S. (2003). Antibody and Genetic Testing in Coeliac Disease. Pathology.

[67] Al-Hussaini A., Eltayeb-Elsheikh N., Alharthi H., Osman A., Alshahrani M., Sandogji I., Alrashidi S., Bashir M.S. (2019). HLA-DQ Genotypes Relative Risks for Celiac Disease in Arabs: A Case-Control Study. J. Dig. Dis..

[68] Pietzak M.M., Schofield T.C., McGinniss M.J., Nakamura R.M. (2009). Stratifying Risk for Celiac Disease in a Large At-Risk United States Population by Using HLA Alleles. Clin. Gastroenterol. Hepatol..

[69] Castro-Antunes M.M., Crovella S., Brandão L.A.C., Guimaraes R.L., Motta M.E.F.A., Silva G.A.P. (2011). da Fequency Distribution of HLA DQ2 and DQ8 in Celiac Patients and First-Degree Relatives in Recife, Northeastern Brazil. Clinics.

[70] Fernández-Cavada-Pollo M.J., Alcalá-Peña M.I., Vargas-Pérez M.L., Vergara-Prieto E., Vallcorba-Gómez-Del Valle I., Melero-Ruiz J., Márquez-Armenteros A.M., Romero-Albillos J.A., Narváez-Rodríguez I., Fernández-de-Mera J.J. (2013). Celiac Disease and HLA-DQ Genotype: Diagnosis of Different Genetic Risk Profiles Related to the Age in Badajoz, Southwestern Spain. Rev. Esp. Enferm. Dig..

[71] Verma A.K., Mechenro J., Monachesi C., Venugopal G., Catassi G.N., Lionetti E., Ramakrishna B.S., Catassi C. (2022). Distribution of Celiac Disease Predisposing Genes HLA-DQ2 and HLA-DQ8 in the Native Population of Southern India. Indian J. Gastroenterol..

[72] Mohta S., Rajput M.S., Ahuja V., Makharia G.K. (2021). Emergence of Celiac Disease and Gluten-Related Disorders in Asia. J. Neurogastroenterol. Motil..

[73] Verma A.K., Singh A., Gatti S., Lionetti E., Galeazzi T., Monachesi C., Franceschini E., Ahuja V., Catassi C., Makharia G.K. (2018). Validation of a Novel Single-Drop Rapid Human Leukocyte Antigen-DQ2/-DQ8 Typing Method to Identify Subjects Susceptible to Celiac Disease. JGH Open.

[74] Makharia G.K., Catassi C. (2019). Celiac Disease in Asia. Gastroenterol. Clin..

[75] Poddighe D., Rakhimzhanova M., Marchenko Y., Catassi C. (2019). Pediatric Celiac Disease in Central and East Asia: Current Knowledge and Prevalence. Medicina.

[76] Sharp S.A., Jones S.E., Kimmitt R.A., Weedon M.N., Halpin A.M., Wood A.R., Beaumont R.N., King S., van Heel D.A., Campbell P.M. (2020). A Single Nucleotide Polymorphism Genetic Risk Score to Aid Diagnosis of Coeliac Disease: A Pilot Study in Clinical Care. Aliment. Pharmacol. Ther..

